# Comparing outcome and recanalization results in patients with anterior circulation stroke following endovascular treatment with and without a treatment with rt‐PA: A single‐center study

**DOI:** 10.1002/brb3.974

**Published:** 2018-04-16

**Authors:** Mohamed Al‐Khaled, Toralf Brüning, Carina Gottwald, Florian Roessler, Georg Royl, Thomas Eckey

**Affiliations:** ^1^ Department of Neurology University of Lübeck Lübeck Germany; ^2^ Department of Neurology Bundeswehrkrankenhaus Hamburg Germany; ^3^ Department of Neurology University of Giessen Giessen Germany; ^4^ Department of Neuroradiology University of Lübeck Lübeck Germany

**Keywords:** acute ischemic stroke, anterior circulation, endovascular treatment, IV rt‐PA, mechanical thrombectomy, mortality, outcome

## Abstract

**Objectives:**

Endovascular treatment (ET), in addition to a therapy with intravenous recombinant tissue plasminogen activator IV rt‐PA in patients with acute ischemic stroke, has been found to improve outcome. However, data about ET in patients who have not received therapy with rt‐PA due to contraindications for IV rt‐PA are sparse. Comparison of ET with IV rt‐PA versus ET alone in patients with stroke is done using a proximal intracranial arterial occlusion (internal carotid artery, middle cerebral artery (M1‐Segment)).

**Methods:**

During a 5‐year period (2011–2016), 236 patients (mean age, 69 ± 14 years; 46% women; median NIHSS score 13 ± 5) who were treated at the Department of Neurology and Neuroradiology at the University of Lübeck, undergoing ET with or without IV rt‐PA were included and analyzed.

**Results:**

A total of 144 patients (61%) underwent ET + IV rt‐PA, and 92 patients (39%) underwent ET only. The ET with IV rt‐PA is associated with a higher rate of favorable functional outcomes (mRS≤2) at discharge from hospital (51.4% vs. 23.1%, *p < *.001) and lower rate of in‐hospital mortality (9% vs. 19.6%, *p = *.019) and symptomatic intracerebral hemorrhage [sICH] (2.1% vs. 8.7%; *p *=* *.019) compared to ET, whereas the modified treatment in cerebral infarction score (mTICI) did not differ between the groups.

In the adjusted logistic regression analysis, the ET + IV rt‐PA was associated with an increased probability of favorable functional outcome (OR, 4.3; 95% confidence interval [CI], 2.2–8.5; *p *<* *.001). For the in‐hospital mortality (OR, 0.74; 95% CI, 0.29–1.9; *p = *.76) and sICH (OR, 0.3; 95% CI, 0.07–1.2; *p *=* *.09), no differences were found.

**Conclusion:**

Recanalization results after endovascular treatment are not relevantly improved in patients receiving rt‐PA. However, an additional therapy with IV rt‐PA has a positive impact on functional outcome.

## 1 INTRODUCTION

The efficacy of endovascular treatment (ET) versus medical treatment alone in patients with stroke caused by occlusion of the middle cerebral artery or the internal carotid artery has been proven in several randomized studies (Berkhemer et al., 2015; Campbell et al., 2015; Goyal et al., 2015a; Molina et al., 2015; Saver et al., 2015). The use of mechanical thrombectomy has gradually spread since 2004, when the first retriever device was approved for endovascular treatment in stroke patients (Smith et al., 2005). In subsequent years, a number of various endovascular device systems (Penumbra aspiration, stent retriever) have been approved (Nogueira et al., 2012; Penumbra Pivotal Stroke Trial I, 2009; Saver et al., 2012).

Several recent randomized trials of endovascular treatment for AIS showed reduced disability among stroke patients who were treated with ET in addition to standard IV rt‐PA (Berkhemer et al., 2015; Campbell et al., 2015; Goyal et al., 2015a; Molina et al., 2015; Saver et al., 2015). MR Clean, IMS III, SWIFT PRIME, and ESCAPE were designed to determine safety and efficacy of endovascular treatment in AIS patients in addition to standard IV rt‐PA (0.9 mg alteplase per kilogram of body weight within a 4.5‐hr time window after onset of stroke; intervention group) compared to continued alteplase alone (Berkhemer et al., 2015; Broderick et al., 2013; Goyal et al., 2015a). Recent studies have found similar benefits of outcomes by the comparison of ET alone vs. ET with rt‐PA bridging (Broeg‐Morvay et al., 2016; Coutinho & Pereira, 2017; Coutinho et al., 2017; Rai et al., 2017; Tsivgoulis et al., 2016; Weber et al., 2017).

The aims of the present study are to compare the outcomes in patients who received ET in addition to standard IV rt‐PA therapy versus those who received only ET due to contraindications for IV rt‐PA.

## 2 METHODS

### 2.1 Study design

Between July 2011 and December 2016, all consecutive patients with AIS in the anterior circulation who underwent mechanical thrombectomy with or without IV rt‐PA at the departments of Neurology, University of Lübeck were included in this single‐center study and analyzed. Clinical severity was assessed based on the National Institutes of Health Stroke Scale [NIHSS] score. In accordance with national and international stroke guidelines, patients received IV rt‐PA at a dose of 0.9 mg per kilogram of body weight within 4.5 hr after onset of anterior circulation ischemic stroke. In patients with a wake‐up stroke or uncertain time frame since symptom onset IV rt‐PA could be administered nonetheless depending on image morphologic aspects in mandatorily required magnetic resonance imaging (MRI) or CT‐based Penumbra imaging with CBF/CBF mismatch in these cases. All included patients had occlusion of either the distal internal carotid artery or the first segment of the middle cerebral artery.

The primary outcomes of the study were the in‐hospital mortality and a favorable outcome at discharge from hospital, as measured on the modified Rankin Scale (mRS ≤2) (Bruening & Al‐Khaled, 2015). In addition, we assessed complications; symptomatic intracerebral hemorrhage (sICH) after treatment during hospitalization which was diagnosed according to ECASS III (Hacke et al., 2008). All patients included in the study were admitted to the stroke unit or intensive care unit (if an early tube removal was not possible) after intervention. All patients were treated by stroke neurologists and neuroradiologists. The study was part of the stroke registry as a benchmark project. The registration of stroke patients in the stroke registry is obligatory. Baseline and sociodemographic characteristics, such as gender, age, comorbid conditions, neurological deficits on admission, incident complications, and clinical findings, were identified from clinical records and the hospital information system and are presented in Table 1. The contraindications for the treatment with IV rt‐PA among patients who underwent only ET are listed in Table 2.

**Table 1:** comparison of baseline data and treatment procedures between patients who underwent ET + IV rt‐PA and those who underwent ET only

Baseline characteristics, treatment procedures, and outcomes	Treatment		*p* value
	ET + IV rt‐PA (*n *= 144)	ET only (*n *= 92)	
Age, mean (SD)	69 (13)	68.7 (14)	.8
Female sex (%)	82 (57)	46 (46)	.3
NIHSS, median, points (IQR)	13 (10–17)	13 (10–17)	.5
ASPECTS, median	8 (7–9)	8 (6–9)	0.3
Door to needle, median, minutes (IQR)	28 (22–57)	‐	
SOI, median, minutes (IQR)	204 (155–262)	200 (151–248)	.24
door to groin, median, minutes (IQR)	88 (55–103)	83 (60–103)	.44
Groin to reperfusion, median, minutes (IQR)	50 (29–93)	52 (21–129)	.19
Imaging to intervention, median, minutes (IQR)	63 (40–100)	68 (45–90)	.7
Wake up stroke	21 (15)	22 (24)	.06
In‐hospital stroke	9 (6)	24 (26)	<.001
Transported from other hospitals	52 (36)	32 (35)	.7
Medical history			
Previous stroke	36 (26)	32 (36)	.1
Atrial fibrillation	66 (47)	50 (54)	.3
Hypertension	101 (72)	71 (78)	.3
Diabetes mellitus	31 (22)	18 (20)	.7
Hyperlipidemia	51 (36)	36 (40)	.6
Previous smoking	22 (16)	16 (18)	.7
Coronary heart disease	30 (21)	22 (24)	.6
Premedication			
Statins	29 (22)	29 (33)	.05
Beta blocker	71 (53)	47 (55)	.8
Angiotensin‐converting enzyme inhibitor	41 (31)	25 (29)	.8
Antiplatelet treatment	50 (37)	42 (48)	.08
Findings in initial CT scan and Angiography			
Early signs of ischemia	86 (61)	62 (67)	.3
Dense artery sign	73 (51)	44 (48)	.6
Middle cerebral artery occlusion	132 (92)	82 (89)	.5
Affected side			
Left	76 (33)	52 (22)	.7
Right	65 (28)	40 (17)	
Angiographic outcome (mTICI)			
0	4 (3)	4 (4)	.2
1	1 (0.7)	2 (2)	
2a	105 (73)	58 (63)	
2b	2 (1)	5 (5)	
3	31 (22)	23 (25)	
Hospitalization, median, days (IQR)	11 (8–16)	13 (8–19)	.8
Treatment during hospitalization			
ICA stenting	31 (22)	22 (24)	.7
Phenprocoumon	6 (4)	6 (7)	.09
DOAC	25 (18)	10 (11)	.07
Aspirin	67 (48)	30 (33)	.028
Clopidogrel	28 (20)	20 (22)	.12
Statins	113 (80)	58 (64)	.013
Primary outcomes			
In‐hospital mortality	13 (9)	18 (20)	.019
Good Outcome (mRS0‐ ≤ 2)	73 (52)	21 (23)	<.001
Complications			
sICH	3 (2)	7 (8.7)	.019
Pneumonia during hospitalization	72 (51)	45 (49)	.8

IQR, interquartile range; NIHSS, National Institutes of Health Stroke Scale; DTN, door to needle time; SOI, symptom onset to intervention time; DOAC, direct oral anticoagulants (Dabigatran, Rivaroxaban, Apixaban); sICH, symptomatic intracerebral hemorrhage.

**Table 2:** Contraindications for intravenous treatment with rt‐PA in patients received only ET

Contraindications	*N* (%)
Symptom onset to expected IVT >4.5 hr (including unknown Symptom onset time)s	32 (11)
Demarcated infarction	22 (7.5)
Expanded early signs of infarction	8 (2.7)
History of recent surgery	13 (4.5)
History of recent infarction	10 (3.4)
Recent trauma or fracture	5 (1.7)
History of recent bleeding	9 (3.1)
Peri‐interventional during coronary angiography	1 (0.3)
Thrombus occurred during angiography	2 (0.6)
Oral anticoagulation therapy	41 (14.5)

IVT indicates intravenous thrombolysis.

### 2.2 Endovascular treatment

Following their admission to hospital, patients with stroke underwent a computed tomography (CT) scan of the brain with CT angiography. The decision to carry out ET was made by a stroke neurologist and a neuroradiologist. All ETs were performed as standard procedures under general anesthesia via a transfemoral approach. The majority of patients were treated with stent retrievers in combination with proximal or distal aspiration, only very few by direct aspiration with the Penumbra System^®^. The most commonly used stent‐retriever in our study was the Trevo^®^‐Stent (Stryker), which has been chosen in majority of all thrombectomies. Other retrievers that have been used were the Solitaire Device (ev3), the ReViveSE^™^ Device (Codman), and the 3D Separator (Penumbra). Depending on the anatomy and concomitant pathologies (e.g., carotid artery stenosis), thrombectomies were either performed with proximal aspiration via an 8F balloon guide catheter (Cello^®^, ev3) or distal aspiration during stent retrieval with 5MAX, 5MAX ACE (Penumbra) or Sofia^®^ (MicroVention) distal access catheter. The Penumbra aspiration pump was used to ensure continuous reverse flow, respectively, flow arrest during clot retrieval. The average waiting time for clot integration into the struts was 3 min. The success of the procedure has been assessed by modified treatment in cerebral ischemia (mTICI)‐Score.

### 2.3 Statistics

We used the Statistical Product and Service Solutions (SPSS) program (version 22) to analyze the data. The data were described with mean and SD values for continuous variables, median and interquartile range (IQR) values for scores, and absolute numbers and percentages for nominal and categorical variables. We performed a chi‐square test to determine the correlation between categorical variables, a *t* test between continuous variables, and a Mann–Whitney test between scores. Logistic regression was carried out to estimate the odds ratios (ORs) for in‐hospital mortality and favorable outcome (mRS ≤2). The logistic regression was adjusted for age, sex, NIHSS score at admission, and medical history. The variables such as sex, age, arterial hypertension, diabetes mellitus, hypercholesterolemia, previous stroke, and atrial fibrillation were entered into the logistic regression model. A *p* value <.05 was considered significant.

## 3 RESULTS

Of 236 patients (mean age, 69.0 ± 13 years; 46% women), 144 patients received ET in addition to medical therapy with IV rt‐PA (ET + IV rt‐PA), whereas 92 patients underwent ET only due to contraindications for IV rt‐PA.

A comparison of baseline characteristics at admission between the groups is shown in Table 1.

**Figure 1:**
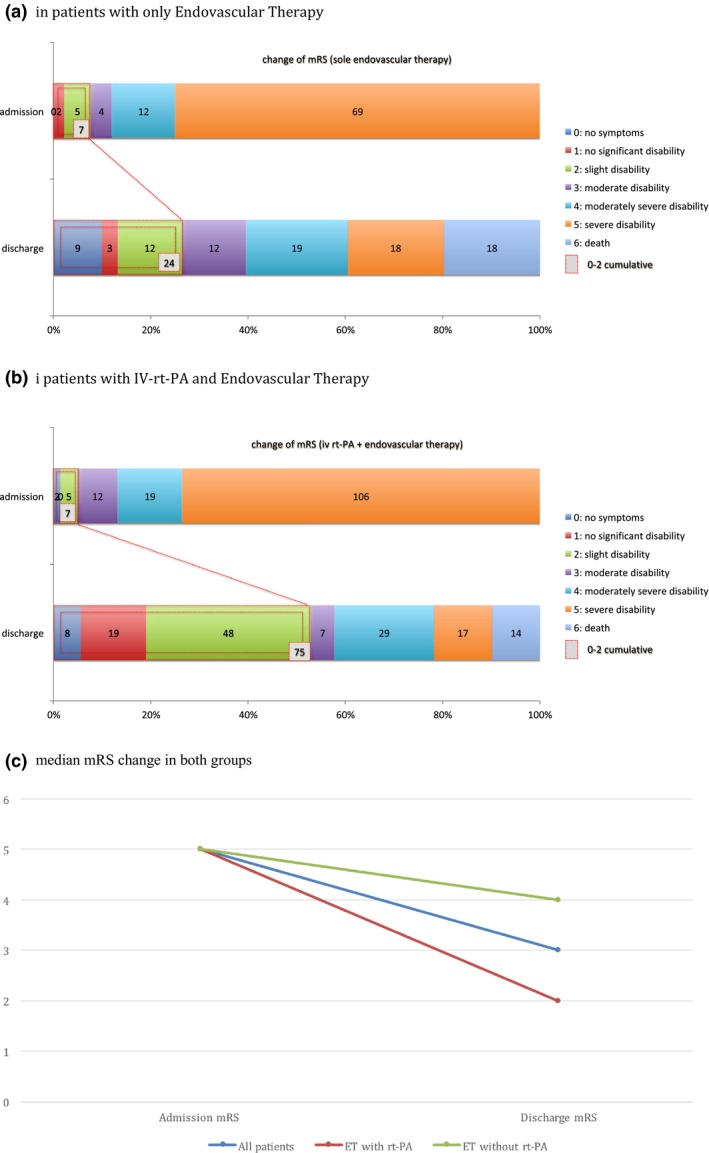
Change of mRS from admission to discharge as well as a comparison of mRS score between admission and discharge. (a) In‐patients with only Endovascular Therapy. (b) In‐patients with IV‐rt‐PA and Endovascular Therapy. (c) Median mRS change in both groups

The parameters leading to a contraindication for IV rt‐PA treatment are shown in Table 2. The most common reasons were pre‐treatment with oral anticoagulants (14.5%), a prolonged time window (time from symptom onset possibly or definitely >4.5 hr, 11%), and a visible infarct demarcation or suggestive of a subacute infarction with an onset >4.5 hr. Nevertheless, 50% of 42 patients with wake up stroke i.v. rt‐PA was administered off‐label based on penumbra imaging with perfusion‐weighted CT or MRI. The median mRS at admission was 5 (IQR = 4–5) among the whole cohort and decreased significantly (*p *<* *.001) after treatment to 3 (IQR = 2–5) at discharge. The change of mRS from admission to discharge is shown in Figure 1, by dividing the group with rt‐PA versus without. In both groups, we found a significant decrease in mRS from admission to discharge; among patients who received rt‐PA therapy (from 5 to 2; *p *<* *.001) and among patients with only ET (from 5 to 4; *p *<* *.001).

**Figure 2:**
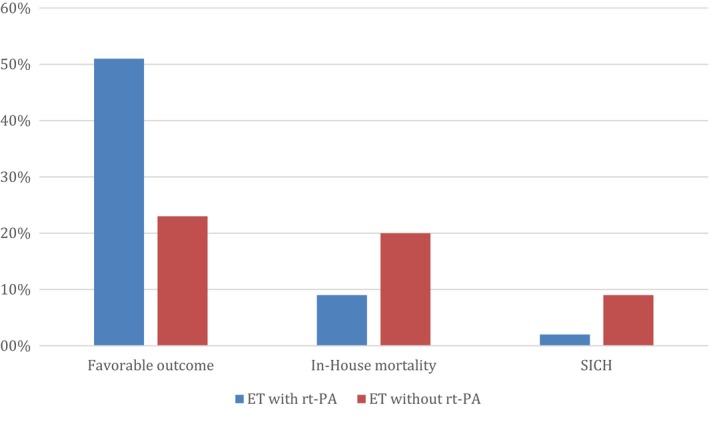
Comparison of favorable outcome, in‐House mortality and symptomatic intracerebral hemorrhage (SICH) between ET with rt‐PA versus without

By comparing the two groups, with rt‐PA vs. without, the rate of favorable functional outcome at discharge from hospital (mRS ≤ 2) was higher (51.4% versus 23.1%, respectively; *p *<* *.001) and in‐hospital mortality was lower (9% versus 19.6%, respectively; *p *=* *.019) among patients who underwent ET + IV rt‐PA versus those who underwent ET only (Figure 2).

Furthermore, the frequency of sICH was lower in patients with IV rt‐PA (2.1% versus 8.7%; *p *=* *.019) compared to the patients who underwent ET only (Figure 2). The recanalization of the anterior circulation determined with mTICI did not show a significant difference between groups (Table 1).

Adjusted logistic regression revealed that treatment with ET + IV rt‐PA was associated with an increased rate of favorable functional outcome at discharge (OR, 4.3; 95% CI, 2.2–8.5; *p *<* *.0001), whereas no differences were found among the in‐hospital mortality (OR, 0.74; 95% CI, 0.3–1.9; *p *=* *.76) and rate of sICH (OR,0.3; 95% CI, 0.07–1.2; *p *=* *.09).

A difference in the recanalization rate assessed by mTICI scores was found similar in both groups (Table 1). During a median hospitalization of 11 days, the occurrence of stroke‐related pneumonia did not differ between patients who underwent ET + IV rt‐PA and those who underwent ET only (51% versus 49%, respectively; *p *=* *.8).

## 4 DISCUSSION

In the present study, 61% of AIS patients received an intervention with ET in addition to IV rt‐PA therapy, whereas 39% underwent ET only. For patients who underwent ET only, this was the only option for stroke therapy because of existing contraindications of treatment with IV rt‐PA (Table 2). Of these patients, 18% presented with wake‐up stroke, and 17% were medicated with oral anticoagulants.

The severity of stroke and the most common baseline characteristics did not differ between the two groups. In addition, the median time of 200 min between symptom onset and beginning of intervention (groin puncture and placement of thrombectomy device) was comparable to those of previous investigations(Campbell et al., 2015; Saver et al., 2015), and showed no difference between patients who underwent ET + IV rt‐PA and those who underwent ET only. About 36% of patients primarily presented to other hospitals and then they were immediately transported to our hospital to receive extended stroke care. The rate of transferred patients was similar in both treatment groups.

Among the entire cohort, an improvement of neurological deficits measured on the mRS score was found, whereas the rate of improvement of the neurological deficits was notably higher in the patients group who received additional rt‐PA treatment. Implementation of rt‐PA was associated with a better outcome (OR, 4.3). Comparable to other studies, neither in hospital mortality nor sICH did show any difference in the logistic regression analysis (Al‐Khaled, Eggers, & Qug, 2014; Al‐Khaled, Matthis, & Eggers, 2014; Berkhemer et al., 2015; Bruning & Al‐Khaled, 2015).

The finding of a better outcome in patients with additional i.v. thrombolysis has to be interpreted with caution. All contraindications to i.v. thrombolysis (extended time window, anticoagulation, surgery) are likely to contribute to worsening of outcome in patients with acute stroke. In addition, recent data suggest a better outcome after endovascular treatment in patients receiving conscious sedation compared to patients receiving general anesthesia (Campbell et al., 2018). The patients in our study were all treated under general anesthesia. However, a relevant systematic group bias is improbable because general anesthesia would affect both groups.

Strengths of the present study are its monocenter setting, in that all patients received treatment from the same medical team and the first study to compare the outcomes of patients who underwent ET + IV rt‐PA and those who underwent ET only due to contraindications for rt‐PA. In previous studies, notably the study by Goyal et al. (2015b), approximately, 25% of patients did not receive treatment with IV alteplase. Moreover, in the Multicenter Randomized Clinical Trial of Endovascular Treatment for Acute Ischemic Stroke in the Netherlands (MR CLEAN) investigation, 10% of patients who had undergone a thrombectomy did not receive medical treatment with rt‐PA. In both studies, a reduced benefit associated with the performance of only ET was not found (Berkhemer et al., 2015; Goyal et al., 2015a). However, a recent meta‐analysis found a favorable outcome in patients with additional i.v. Thrombolysis (Mistry et al., 2017). Our study adds further support to that found by the meta‐analysis by Mistry et al., 2017 and to the recent study by Nogueira et al. (2018) showing better outcomes among stroke patients who underwent ET within 6–24 hr after symptom onset.

Our study also has several limitations: Its design was not randomized. Thus, the finding of a better outcome in patients with additional i.v. thrombolysis has to be interpreted with caution. All contraindications to i.v. thrombolysis (extended time window, anticoagulation, surgery) are likely to contribute to a worsening of outcome in patients with acute stroke.

Despite these limitations, it is a single‐center real‐life study showing that medical treatment with IV rt‐PA before thrombectomy may improve functional outcome compared with the interventional treatment alone.

## CONFLICT OF INTERESTS

None
